# Efficient Unsupervised Classification of Hyperspectral Images Using Voronoi Diagrams and Strong Patterns

**DOI:** 10.3390/s20195684

**Published:** 2020-10-05

**Authors:** Laura Bianca Bilius, Ştefan Gheorghe Pentiuc

**Affiliations:** 1 The Machine Intelligence and Information Visualization Lab (MintViz), Integrated Center for Research, Development and Innovation in Advanced Materials, Nanotechnologies, and Distributed Systems for Fabrication and Control (MANSiD) Research Center, “Stefan cel Mare” University of Suceava, 720229 Suceava, Romania; 2 Computers and Information Technology Department, “Stefan cel Mare” University of Suceava, 720229 Suceava, Romania

**Keywords:** Voronoi diagrams, Parafac decomposition, hyperspectral images, classification, abundances map, strong patterns, consensus partition

## Abstract

Hyperspectral images (HSIs) are a powerful tool to classify the elements from an area of interest by their spectral signature. In this paper, we propose an efficient method to classify hyperspectral data using Voronoi diagrams and strong patterns in the absence of ground truth. HSI processing consumes a great deal of computing resources because HSIs are represented by large amounts of data. We propose a heuristic method that starts by applying Parafac decomposition for reduction and to construct the abundances matrix. Furthermore, the representative nodes from the abundances map are searched for. A multi-partition of these nodes is found, and based on this, strong patterns are obtained. Then, based on the hierarchical clustering of strong patterns, an optimum partition is found. After strong patterns are labeled, we construct the Voronoi diagram to extend the classification to the entire HSI.

## 1. Introduction

Satellite hyperspectral sensors are in a growing stage of development and are used in many applications, such as Earth observations, remote sensing and urban mapping. Remote sensing is a technology through which it is possible to acquire information at the Earth’s surface without having direct contact with the elements. The absorption and emission properties of electromagnetic radiation vary from existing material on the Earth’s surface in the visible to near-infrared and short-wave infrared ranges [1,2]. Remote sensing technology takes images in various wavelengths that then are transmitted to the Earth through telecommunication [3]. The spectral signature of material is a powerful tool to identify materials that are present in an area [1]. The information acquired from satellites or aircraft are multispectral and hyperspectral data [4].

Hyperspectral image (HSI) acquisition has developed in recent years along with the remote sensing techniques used to study the data. According to to [1], the platforms for hyperspectral images are airborne, such as AVIRIS, HYDICE, CASI or HYMAP, and spaceborne, such as Hyperion, HYSI, MODIS or CHRIS. Nevertheless, HSI presents advantages regarding spectral resolution, area coverage and spectral bandwidth. Each pixel has all the radiance values for the corresponding wavelength collected by the sensor [1]. HSIs may have various spectral and spatial resolutions. For example, the hyperspectral images provided by satellites such as Hyperion on EO-01 covers a 400 nm to 2500 nm spectral range and has a spatial resolution of 30 m, while GeoEye-1 covers a 450 nm to 920 nm spectral range and has a spatial resolution of 1.8 m [5].

Hyperspectral and multispectral images offer us the possibility to analyze objects on the Earth’s surface. In comparison, hyperspectral images have a high spectral resolution but low spatial resolution; meanwhile, multispectral images have a low spectral resolution but high spatial resolution [6]. Another difference is that multispectral images acquire spectral signals with fewer discrete values of wavelength bands and hyperspectral images acquire quasi-continuous values (the spectral is less than 10 nm) [6,7]. Taking into account that the spectral information is richer for hyperspectral images, this offers more availability to see unknown aspects of objects.

A hyperspectral image (HSI) is a three-dimensional image cube that is composed of hundreds of spatial images; more precisely, each pixel is a one-dimensional vector that contains a continuous spectrum measured at different wavelengths. Thus, HSI has two spatial dimensions and one spectral dimension [8]. The spectral bands that correspond to the spectral dimension are unique and describe the elements present on the Earth’s surface [9].

Nevertheless, real-world hyperspectral datasets often present problems that affect classification accuracy. Dataset sizes have developed a great deal in recent years but also present real problems, such as cloud-covered areas and their spectral and spatial resolution. Except for real-world challenges, big data also affects the data analysis because of the demand of huge computational resources and the presence of mixed pixels [10]. The goal is to find an algorithm to study the data with a fast and accurate technique.

Tensor decomposition is used to solve classification problems for big data. Phan et al. [11] proposed a model for reduction, feature extraction and classification problems based on Tucker decomposition. Jouni et al. [12] proposed a method to create a matrix with a low-dimensional space with the help of CP (canonical polyadic) decomposition. Another approach of tensor decomposition was proposed by Makantasis et al. [13]. The proposed model adopts canonical decomposition as a linear combination of weight parameters. The model was used for data classification and analysis. A dimensionality reduction was also proposed by Liu et al. [14] by combining the manifold structure of high-dimensional data and the linear nearest neighbor relationship.

Considering spectral unmixing, various algorithms have been proposed or analyzed in the literature. Ibarrola Ulzurrun et al. [15] studied spectral unmixing models such as LMM (Linear Mixing Model), FCLSU (Fully Constrained Least Squared Unmixing), SCLSU (Scaled Constrained Least Squares Unmixing), ELMM (Extended Linear Mixing Model) and RELMM (Robust Extended Linear Mixing Model), with the latter giving the best accuracy value.

In the literature, there are also authors such as Villa et al. [16] who have approached the study of mixed pixels. Their paper proposed a method based on soft classification techniques and spectral unmixing algorithms to solve the problem of mixed pixels. Plaza et al. [17] proposed a new approach of the unsupervised classification of mixed pixels using extended morphological profiles and derivative analysis. According to the experimental results, the proposed method offered applicability in mixed pixel analysis and so can be used on hyperspectral images with noise and low spatial resolution. Considering the availability of hyperspectral data and the challenges that come with it, continuous attention must be paid to the possibility of studying the elements from an area of interest without having direct contact.

HSI classification involves labeling pixels in an image with class labels. Each class corresponds to the materials in the image, and the learning process is a supervised one based on expert knowledge, as in [18]. Among the most effective supervised learning methods are the Support Vector Machine [19] or those based on Random Multi-Graphs [9] that use combined spectral and spatial features. Unsupervised classification aims to label the pixels of an HSI without prior information. Both contexts can be addressed through deep learning. An analysis of the use of this technique in a passive imaging remote sensing platform may be found in [20].

Tensor decomposition offers the possibility to study the data while avoiding mixed pixel problems and a high execution time. Another advantage of tensor decomposition is the possibility to approach an unsupervised classification, because the absence of ground truth for some data sets makes a supervised classification impossible. Additionally, according to Phan et al. [11], tensor decomposition methods such as Parafac and Tucker decomposition are used for feature extraction and classification problems for big data.

Voronoi diagrams are used in a number of areas such as astronomy, cartography, geography, mathematics, pattern recognition, robotics, statistics, image processing and so on [21]. Voronoi diagrams have been used in pattern recognition in combination with computational geometry methods and neural networks to design a classifier for the objects from a given dataset [22]. Furthermore, Voronoi diagrams are used for image compression and for creating mosaics [23] or in the image segmentation of various geospatial images [24]. William et al. [25] studied a method called V-synth to sample imbalanced data sets using Voronoi diagrams, with the authors proposing in future work to improve their results by determining accurate boundaries and synthetic points.

In this paper, we propose a classification method using Parafac decomposition, Voronoi diagrams and strong patterns without having previous knowledge of the image content. The construction of the Voronoi Diagram is based on the choice of the most representative nodes from the abundances matrix. The abundances matrix is constructed by multiplying the first two factor matrices which correspond to the spatial dimensions from Parafac decomposition. The Voronoi nodes are searched from the abundance map, and we obtain a multi-partition by using unsupervised algorithms such as K-means, hierarchical clustering and mean-shift. We build the association partition matrix to identify strong patterns. The strong pattern data are classified using hierarchical clustering, and then we construct the corresponding Voronoi diagram.

## 2. Methodology

The unsupervised processing of hyperspectral images consumes a large amount of computing resources, primarily due to the large size of the images. These are represented by three-dimensional matrices in which the number of elements in each dimension is of the order of hundreds. For these dimensions, the execution of a clustering algorithm from the scikit-learn library machine learning method in Python [26,27] may require a time on the order of minutes. The application of a single unsupervised clustering algorithm is not sufficient in all cases. Even the application of the same algorithm with different values for some parameters or various ways of initializing the starting solution can lead to several different partitions being obtained.

Because there is not a single general algorithm that is applicable in any situation, it is necessary to try to apply several unsupervised classification algorithms that will produce several partitions of the pixels in the image. With these partitions, the problem of selecting a single partition to be the solution of the problem arises. Finding a consensus partition of these partitions—as it is called in [28]—or a central partition—as it was named in [29]—is an NP-difficult problem. Solving this in an efficient time involves the application of a heuristic. Thee repeated application of several classification algorithms leads to the consumption of a computation time that varies polynomially with the number of pixels in the image. All these require finding an efficient solution for unsupervised image classification so that the occupied memory and the total execution time are acceptable for the users.

In the case of low-resolution images, in which a pixel in the image represents an area of a few square meters, it is obvious that the spectrum of that point is the result of different materials existing in that area. Thus, the phenomenon of mixing the spectra appears, and the problem that must be solved is the unmixing of spectra stored in the pixels of the hyperspectral images.

**Figure 1:**
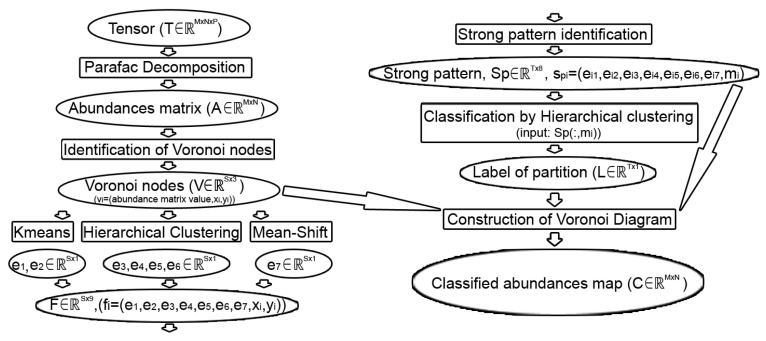
The process flow of the proposed classification method. $T\in R^{M\times N\times P}$—Hyperspectral image; $A\in R^{M\times N}$—the abundances matrix; $V\in R^{S\times 3}$—the Voronoi nodes, where *S* is the number of nodes; $e_{1},e_{2},e_{3},e_{4},e_{5},e_{6},$ and $e_{7}\in R^{S\times 1}$—the label of nodes for each used classification; $F\in R^{S\times 9}$—matrix with partitions and coordinates of nodes; $Sp\in R^{T\times 8}$—the strong pattern, where *T* is the number of strong patterns, and the last column of Sp contains the mean of abundances matrix values of all pixels corresponding to each strong pattern; $L\in R^{T\times 1}$—the label for each strong pattern after hierarchical classification; $C\in R^{M\times N}$—the classified abundances map after we construct the Voronoi diagram using Voronoi nodes.

The proposed method involves the execution of several steps and is represented by the scheme depicted in Figure 1. The method is based on the tri-linear decomposition of the data array, a subdivision of the abundance map in uniform pixel cells and multiple cluster analysis.

### 2.1. Step 1

The aim of the first step is to reduce the dimensionality of the hyperspectral image model and to unmix the spectrum of pixels. For this task, a tri-linear decomposition method such as Parafac decomposition can be used. In Parafac decomposition, the hyperspectral dataset $T\in R^{M\times N\times P}$ decomposes into a sum of component rank-one tensors and is defined as [30]

(1) $$T\approx \sum_{r=1}^{R}a_{r}\circ b_{r}\circ c_{r}+E\approx 〚A,B,C〛$$

where R=rank(T) is the number of components and $A\in R^{M\times R}$, $B\in R^{N\times R}$ and $C\in R^{P\times R}$ are the factor matrices that contain the vectors from rank-one components [30]. Note that A and B are the factor matrices that correspond to the spatial dimension and C corresponds to the spectral dimension. The sum of the smallest number of rank-one components that generates a tensor is defined as the rank of a tensor [30]. According to Kolda [30], for a tensor $T\in R^{M\times N\times P}$, only the following weak upper bound on its maximum rank is shown:

(2) rank(T)≤min{MN,NP,MP}

We employed tensor decomposition to solve the problem of the presence of mixed pixels in the hyperspectral dataset [31]. In a hyperspectral image, there are pure pixels but also mixed pixels. The spectral signature of a pure pixel is relevant because there is only one element in the corresponding area. Nevertheless, there are mixed pixels present in a hyperspectral image due to the low spectral and spatial resolution. The mixed pixels contain more than one element, and so the spectral signature is not relevant; therefore, the accuracy of the classification will decrease [32]. The mixture models $X_{m}\in R^{K_{m}\times N_{m}}$ for a number $N_{m}$ of pixels is defined as

(3) $$X_{m}=M_{m}S_{m}+\gamma$$

where $S_{m}\in R^{R_{m}\times N_{m}}$ contains all the abundances of all pixels on all endmembers, $M_{m}\in R^{K_{m}\times R_{m}}$ is a spectrum matrix where each column corresponds to the spectrum of an endmember, $K_{m}$ is the number of observed bands, $R_{m}$ is the number of endmembers and corresponding abundances and γ is the additive noise [33]. As we can see in Equation (4), $E\in R^{M\times N}$ is the product between matrices A and B and is the corresponding abundance map (see Equation (1)) [33].

(4) $$T=(A\cdot B^{T})\circ C=E\circ C$$

According to Equation (3), which can be seen as a matrix factorization problem, and Equation (4), there is a link between the linear mixture models and Parafac decomposition, and we can affirm that $E\in R^{M\times N}$ is the abundance map [33]. Spectral unmixing extracts the abundances from HSI, and so the spectral and spatial information is kept in the abundances map with the correlations between pixels [34].

We applied Parafac decomposition on our hyperspectral dataset to reduce the model and obtain the abundances matrix.

### 2.2. Step 2

In this stage, we attempt to reduce the size of the learning set (the set of elements of the abundance matrix). In this process, we start from the idea that if we find a Voronoi diagram that approximates the initial matrix in an acceptable way, then this matrix could be represented by the Voronoi nodes of that diagram. It is obvious that if we apply a series of labels to Voronoi nodes, it will be possible to build a Voronoi diagram that will propagate the labeling of all regions based on the labels of the nodes. If Voronoi nodes are classified, they will have their class labels, and thus the partitioning of the nodes can be extended to the entire image. To find the most representative Voronoi nodes, the heuristic algorithm described below will be applied.

Let $E_{n}\in R^{M\times N}$ be the normalized abundances map. We aim to discretize the matrix $E_{n}$ into subintervals of lengths ϵ:

(5) $$e_{d}\left(i,j\right)=\left[\frac{e_{n}\left(i,j\right)}{\epsilon }\right]\subset N\;,\;e_{d}\left(i,j\right)\in E_{d},\;e_{n}\left(i,j\right)\in E_{n}$$

We construct a new matrix F that we will use to find the Voronoi nodes. Firstly, the matrix $E_{d}$ is arranged into a set $Z\in R^{MN}$. We construct the set $S_{u}\in R^{Q}$ that contains the unique values from *Z* in ascending order. We construct the matrix $F\in N^{M\times N}$ by associating the values of pixels from $E_{d}$ with the index of pixel values from $S_{u}$:

(6) $$\begin{array}{c} Z=\left\{e_{d}\left(i,j\right)\in E_{d}\right\}=\left\{z_{1},z_{2},\ldots ,z_{k}\right\},\;\forall i,\;j,\;i=\bar{1,M},\;j=\bar{1,N},\;k=\bar{1,M\times N}\\ S_{u}=\left\{s_{u_{1}},s_{u_{2}},\ldots ,s_{u_{q}}\right\},\;s_{u_{i^{\prime }}}<s_{u_{j^{\prime }}},\;i^{\prime }<j^{\prime },\;\forall i\prime ,\;j\prime ,\;S_{u}\subset Z\\ f\left(i,j\right)=t,\mathrm{if}\;e_{d}\left(i,j\right)=s_{u_{t}},\;t=\bar{1,q} \end{array}$$

To find the nodes, we divided the matrix *F* into square blocks of dimension *n*. We construct the histogram of each block F(i,j) [35]. The number of bins is described in Equation (7) and the histogram is defined in Equation (8), where $h_{i}$ is the number of pixels that belong to category *i* in the block.

(7) $$n_{\epsilon }=\left[\frac{max\left(E_{d}\right)-min\left(E_{d}\right)}{\epsilon }\right]$$

(8) $$H=[h_{1},h_{2},\ldots ,h_{n_{\epsilon }}]$$

If the maximum value of the histogram for the F(i,j) block is bigger than a threshold value equal to p%*nr, where nr is the number of pixels in the block, we assume that the block F is a pure zone, and we calculate the center of gravity of the block that will be a Voronoi node. The value corresponding to the Voronoi node will be the median value of pixels that belong to the maximum value from the histogram. We defined the median value as in Equation (9). Note that *values* is the set that contains the values of pixels that belong to the maximum value from histogram.

(9) $$v_{m}=\left\{\begin{array}{cc} |values|=2k & values([|values|/2]+1)\\ |values|=2k+1 & values([|values|/2]) \end{array}\right.$$

If the previous condition is not met, we split the block F(i,j) into four cells, and we check again the condition for each new cell. If the condition is not met, this process is repeated until the condition is met or the cell contains a single pixel, which will be a Voronoi node:

(10) $$h_{max}=argmax\left(H\right):\left\{\begin{array}{cc} if & H(h_{max)}\geq p*nr/100\;\mathrm{then}\;n_{vor}\left(x_{g},y_{g}\right)\\ else & \mathrm{we}\;\mathrm{split}\;\mathrm{the}\;\mathrm{block}\;\mathrm{in}\;\mathrm{four}\;\mathrm{and}\;\mathrm{repeat}\;\mathrm{the}\;\mathrm{steps}. \end{array}\right.$$

### 2.3. Step 3

Once the set of nodes is defined, they are classified using many clustering algorithms, such as K-means, mean-shift or hierarchical clustering. For hierarchical clustering, for example, the distance between clusters was computed using four different methods, such as the unweighted average distance, centroid distance, farthest distance and inner squared distance, and for K-means, the initial clusters are chosen to speed up the convergence, and for the second computation the initial centers are chosen randomly [27,36]. All classifiers may computed for various number of classes.

For each pixel, we concatenate each class that has been classified by used unsupervised classifiers, we construct the matrix of multi-partitions for all pixels, and then we search for the strong patterns [37].

Let $P_{k}=\left(l_{1},l_{2},\ldots ,l_{r}\right)$ be the collection of predictions of a pixel (i,j), where *r* is the number of labels for a pixel (i,j). For all pixels, the corresponding matrix of predictions $P\in R^{S\times r}$ is defined as in Equation (11) [29] and is called the multi-partition matrix:

(11) $$P=(P_{1},P_{2},\ldots ,P_{k},\ldots ,P_{r})$$

We call a strong pattern the subset of patterns—in our case, a pattern is a Voronoi node—that have always been clustered together [29,37]. For a strong pattern Sp(i), we search for the nodes that were clustered together in the multi-partition matrix.

(12) $$Sp=\left[sp_{1},sp_{2},\ldots ,sp_{T}\right],sp_{i}\left(:\right)\neq sp_{j}\left(:\right),\forall i,j=\bar{1,T},i\neq j,sp_{i}\in R^{1\times r},S_{p}\subset P$$

### 2.4. Step 4

Next, we proceed to find a consensus partition for the multi-partition obtained previously. As mentioned above, this task is an NP-difficult problem. Thus, we adopted a heuristic by applying hierarchical clustering to the strong patterns. The number of classes was chosen by analyzing the graph of the elbow method, the dendrogram and true color image. In the end, we construct the Voronoi diagram using the obtained labelled nodes. Using the strong pattern data and multi-partition data, we assign to each partition from the multi-partition data (which correspond to a Voronoi node) the corresponding label obtained from strong pattern classification.

We started from the basic definition of Voronoi diagrams to construct the Voronoi diagram of the abundances map. The Voronoi diagram is defined as the subdivision of a plane into *n* cells, where *n* is given by distinct points $P=\{p_{1},p_{2},\ldots ,p_{n}\}$ in the plane. The property is that any point *q* which lies in a cell $c_{i}$ corresponds to a point $p_{i}$ with respect to Equation (13) for any $p_{j}\in P$ [38]:

(13) $$dist\left(q,p_{i}\right)<dist\left(q,p_{j}\right),\;\forall p_{i},p_{j}\in P,i\neq j$$

Multiple cluster analysis allowed us to detect strong patterns, which later were classified with an unsupervised classifier and from which we constructed the Voronoi diagram.

## 3. Results

### 3.1. Synthetic Data

**Figure 2:**
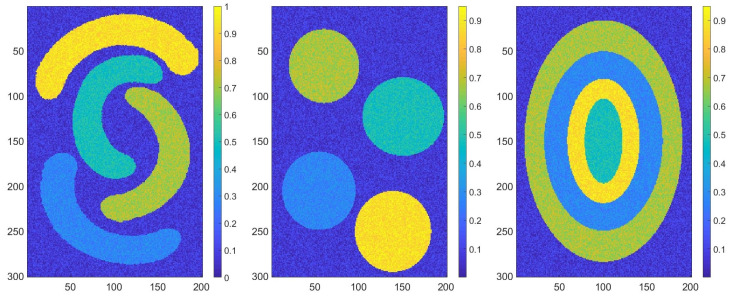
Synthetic data sets.

**Figure 3:**
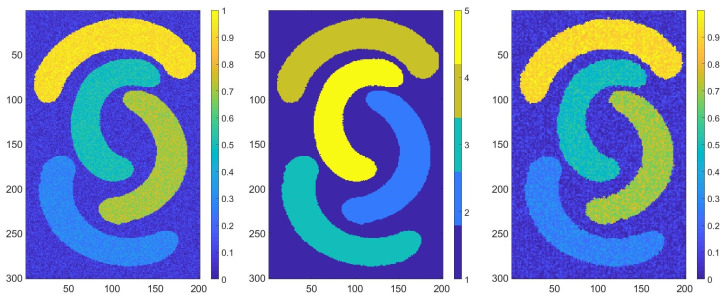
Synthetic data set (**left**), the corresponding ground truth (**center**) and the corresponding Voronoi diagram (**right**).

We tested our method using synthetic datasets (see Figure 2). For this, we generated matrices with values from 1 to 5, as shown in Figure 3 (center), that represented the ground truths. Using the previous matrices, we constructed new matrices by replacing each value from the ground truths with a random number. For each number from 1 to 5, we defined an interval to generate a random number from it. The new matrices obtained formed the dataset that we aimed to classify.

We describe the obtained results for one dataset—the matrix $D\in R^{M\times N}$—that can be seen in Figure 3 (left). For all our datasets, M=201 and N=301, and all values are from 0 to 1.

We computed the algorithm to identify the Voronoi nodes for various parameters; more precisely, different values for ϵ and purity. As can be seen in Table 1, if the value of ϵ or the purity increases, the number of Voronoi nodes also increases. The error is calculated as in Equation (14), where $D\in R^{M\times N}$ is the original data and $VD\in R^{M\times N}$ is the diagram obtained after classification.

(14) $$er=\frac{\sum_{i,j=1}^{M,N}\left|D\left(i,j\right)-VD\left(i,j\right)\right|}{\sum_{i,j=1}^{M,N}D\left(i,j\right)}$$

For a better visualization of the results, the construction of the Voronoi diagram using the set of nodes for ϵ=0.01, purity =30 and a dimension of the block of 5 is shown in Figure 3 (right). The number of nodes for this parameter was 25,489 from 201×301.

To construct the multi-partition, we used the classifiers K-means, hierarchical clustering and mean-shift on median values (see Equation (9) of Voronoi nodes as described in Section 2. We used the parameters described in Section 2 for classifiers, and we obtained a matrix of partitions of size 25,489 × 7. In all situations, the number of clusters was 5. We searched for strong patterns and obtained a matrix of size 9×7; thus, we found nine strong patterns. The numbers of nodes in each strong pattern were 2913, 84, 13,645, 460, 2549, 83, 79, 2550 and 3126. Using the strong pattern matrix and multi-partition matrix, we calculated the mean of all median values of Voronoi nodes that belonged to the corresponding strong pattern. Previously calculated mean values were classified using hierarchical clustering (the distance between clusters was computed using unweighted average distance). In the end, the label of each strong pattern obtained after classification was associated with the corresponding partitions from the multi-partition matrix. Following this step, we could perform the final classification of the initial data.

**Figure 4:**
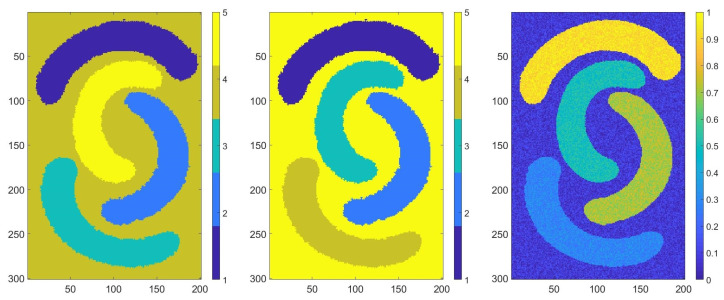
The Voronoi diagram of our dataset classified using seven unsupervised classifiers (**left**) and nine unsupervised classifiers (**center**); the dataset (**right**).

**Figure 5:**
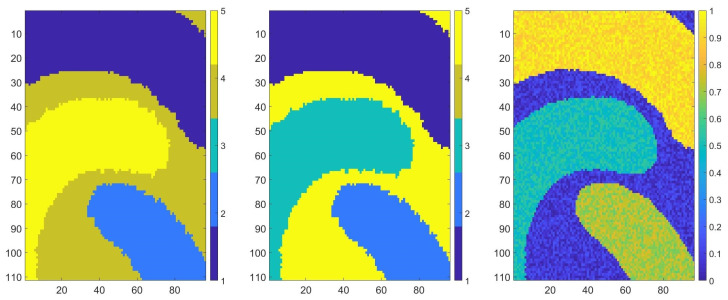
The Voronoi diagram of our dataset classified using seven unsupervised classifiers (**left**) and nine unsupervised classifiers (**center**); the dataset (**right**).

The final result is presented in Figure 4 (left). In Figure 5 (left), we present the result at a detailed scale of a sample from Figure 4 (left). Note that each color represent a class. According to previous results, we decided to add two more classifications to the previous multi-partition matrix with different numbers of classes; for example, we ran K-means with six and seven clusters. Therefore, we obtained a matrix of partitions of size 25,489 × 9. The number of strong patters was 13. We followed the same steps described previously; therefore, the final result is presented in Figure 4 (center), and in Figure 5 (center), the result at a detailed scale of a sample from Figure 4 (center) is shown. In Table 2, the confusion matrices between the final results for both described cases and the ground truth are shown.

### 3.2. Real-World Dataset

We used a real cloudy data set acquired from USGS (science for a changing world) [39]. The data was acquired from the Earth-Observing One (EO-1) satellite and it was provided by Hyperion with a 30 m resolution [40]. Hyperion acquires 220 spectral bands (from 0.357 to 2.576 micrometers) with a 10 nm bandwidth [40].

**Figure 6:**
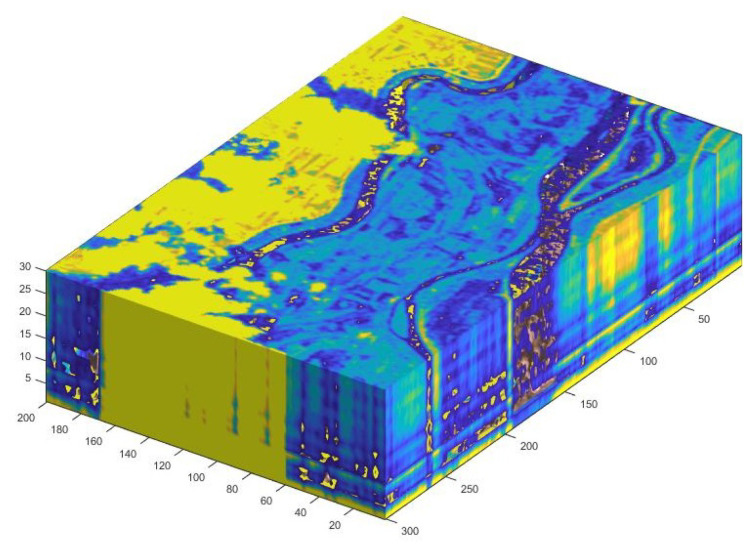
Three-dimensional representation of the data set.

**Figure 7:**
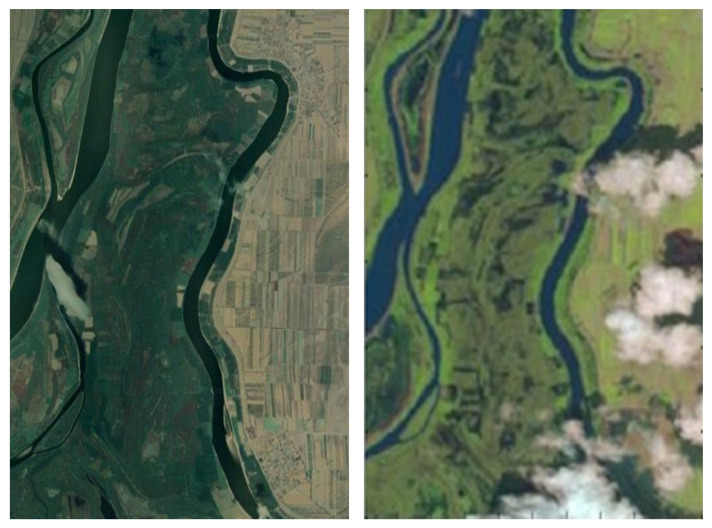
The true color of the Brăila dataset (an area of Brăila’s Small Island; a satellite view available from Google Maps, (**left** [39]), and the true color provided by the EO-1–Hyperion satellite together with the hyperspectral image (**right** [41]; see Figure 6).

The dataset of Brăila—more precisely, Small Island of Brăila from Romania—has 301×201×34 dimensions (see Figure 6), the cloud cover is from 10% to 19%, and according to [39], the data were taken in 2006 (see Figure 7 (right)). We went through each image corresponding to the spectral channel, and we kept only the accurate ones—more precisely, 34 from 220.

The same area of Brăila can be seen in Figure 7 (left) but was taken in 2020, according to Google Maps [41]. The geographic coordinates for Small Island of Brăila are 44.903588 (latitude) and 27.907525 (longitude).

**Figure 8:**
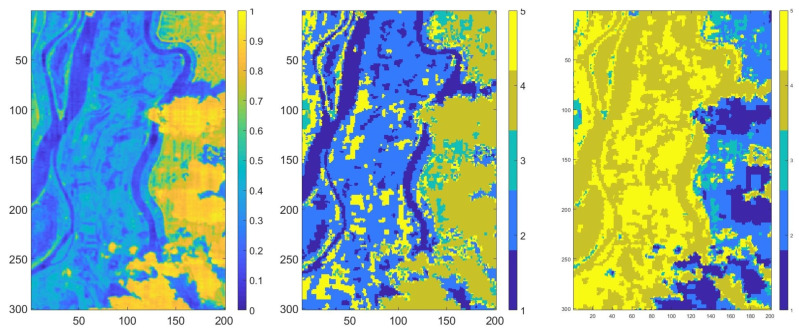
The abundances map of the Brăila dataset (**left**), the classified images using seven unsupervised classifiers (**center**) and nine unsupervised classifiers (**right**).

Firstly, we applied Parafac decomposition to reduce the dataset dimension and extract the constituent spectra and the corresponding abundances. The explained variation of Parafac decomposition using 200 components was 99.18, and the execution time was 6.028 s (see Equation (2). According to Equation (2), we experimented with various numbers of components, and we decided that the most appropriate value in terms of the execution time and explained variations was 200 components. The abundances matrix was constructed by multiplying the first two factor matrices that corresponded to the spatial dimension obtained from Parafac decomposition (see Equation (4)), where each value from the matrix represents the abundance of the corresponding pixel. The abundances matrix was normalized with values from [0,1] and can be seen in Figure 8 (left) [33]. According to Figure 1, after we obtained the abundances map, we searched for the most representative nodes.

We split the matrix into quadratic blocks of dimension *n* to find the most representative nodes. The set nodes (pixels) were chosen following the same algorithm applied in Section 3.1 and described in Section 2.

In Table 3, we show the results of the error and number of nodes for different values for ϵ and purity. In all cases, the block size was 5. To construct the Voronoi set, we used ϵ=0.1, purity =60 and a dimension of the block of 5. The number of nodes for this parameters was 11032 from 201×301 and the error was 6.240 (see Equation (14)).

The multi-partition matrix was constructed after we applied the same unsupervised classifiers as in Section 3.1. For the real dataset, we calculated the number of nodes (%) distributed in each cluster for each classifier and the silhouette scores. Silhouette coefficient values ranged between [−1,1], where a value near to −1 indicated the incorrect assignation of clusters and 1 indicated a clear assignation of clusters [42]. In Table 4, we show the silhouette scores for the unsupervised classifiers we used in our study. The scores indicate a good assignation of classes.

**Figure 9:**
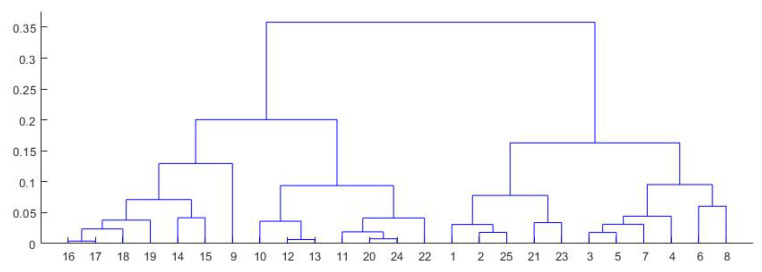
The dendrogram of hierarchical clustering.

**Figure 10:**
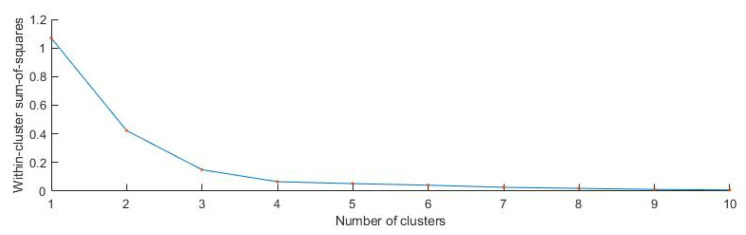
The graph of the elbow method applied to hierarchical clustering.

Next, we searched for the strong patterns in the multi-partition matrix. We found 25 strong patterns after we used seven unsupervised classifiers and 35 after we used nine unsupervised classifiers. To obtain the consensus partition of this multi-partition, we applied a heuristic method: we classified the strong patterns using hierarchical clustering. With the obtained dendrogram, we had to choose the number of clusters for the consensus partition. According to Figure 7, Figure 9 and Figure 10, we created the graph of the elbow method [43]. In Figure 9, the distance between clusters is representative for the five classes. In Figure 10, the elbow is optimal for five clusters. According to these observations, we classified the data into five clusters. In Table 5, we present the distribution of all pixels in each class after the Voronoi diagram was constructed. In Figure 8 (center, right), the final results after we classified the strong patterns into five classes (each color represents a class) and labeled the nodes in the multi-partition matrix with the corresponding class in the strong pattern matrix are presented. The execution time to construct the Voronoi diagram was 7.142 s. Note that the purpose of the colors used is to highlight the classes we obtained.

The proposed method implies a model reduction by applying Parafac decomposition and the classification of a lower amount of data; more precisely, the Voronoi nodes. A strong pattern of data was found from the multi-partition matrix. The strong pattern contains the subset of patterns that were clustered together (in our case, the Voronoi nodes clustered together). The method we applied decreased the computational resources and also resulted in a better classification by using a strong pattern to build the consensus partition. This approach increases the accuracy of classification. The final classification of the abundance map implies the labeling of the Voronoi nodes after seeing the consensus partition and the construction of the Voronoi diagram.

## 4. Conclusions and Discussion

The main purpose of the unsupervised processing of hyperspectral images is the detection of similar areas from a spectral point of view in the image. Because there is no unique algorithm that is applicable in any situation, a possible solution is to create and process a multi-partition in order to find the partition that achieves consensus between the component partitions. Based on this consensus partition, it is possible to provide a classification that is as close as possible to the distribution of materials in the terrain. On the other hand, it was observed that as the size of hyperspectral images increases, the performance of the classification decreases [9]. The unsupervised processing of HSIs requires an efficient use of the computing resources involved in this process, meaning that any attempt to reduce the size of the required memory space and execution time is necessary.

In this paper, we have presented such an approach through a multi-step method that seeks to streamline the process of the unsupervised learning of hyperspectral images. The intervention of a human operator is necessary in the last stage to validate the number of classes of the consensus partition and to establish the physical significance of the classes.

We proposed an efficient method to classify hyperspectral data without having information about the ground truth using Voronoi diagrams and strong patterns (see Figure 1). Firstly, we constructed the abundances matrix using the first two factor matrices obtained after Parafac decomposition. We found the most appropriate Voronoi nodes in order to reduce the number of patterns that had to be classified without supervision.

The results obtained by applying this method on several sets of synthetic data were very good in terms of classification accuracy and from the point of view of the amount of computational resources involved. The method was also applied to a hyperspectral image of an area of Braila’s Small Island—an image acquired from Earth-Observing One (EO-1) satellite that was provided by Hyperion with a 30 m resolution [40]. The multi-partition was obtained by applying three different clustering algorithms that were asked to determine partitions with a variable number of classes and using different execution parameters. The consensus partition of this multi-partition was one with five classes—a number suggested by quantitative methods and based on other information regarding the photographed area.

The processing results were analyzed both from the point of view of the quality of the consensus partition and from the point of view of the physical significance of the classes obtained. The results can be considered to be encouraging for the further development of this method. In further research, we will try firstly to improve the node identification and efficiency of classification in order to be able to apply as many unsupervised classification algorithms as possible for a wider range of possible classes. A larger multi-partition increases the chances of obtaining a more general consensus partition. Secondly, we will try to build the consensus partition as part of an optimization process that aims to maximize the measure of similarity between the dominant spectrum in each class and the existing spectra in a library for materials likely to appear in the area represented by the hyperspectral image.

## Figures and Tables

**Table 1:** The result of errors and number of found nodes using different values for ϵ and purity. The dimension of the blocks was 5.

Epsilon (ϵ)	Purity (p%)	Error	Number of Nodes
0.1	30	18.021%	2534
0.1	50	15.734%	3500
0.1	70	10.095%	24,492
0.1	90	7.141%	36,586
0.01	30	10.116%	25,489
0.01	50	7.490%	35,433
0.01	70	6.505%	38,743
0.01	90	6.490%	38,794
0.001	30	7.050%	36,983
0.001	50	6.601%	38,431
0.001	70	6.488%	38,801
0.001	90	6.488%	38,801
0.0001	30	6.588%	38,447
0.0001	50	6.523%	38,677
0.0001	70	6.488%	38,801
0.0001	90	6.488%	38,801

**Table 2:** The confusion matrices between the final classification of the entire dataset and the initially generated label using seven unsupervised classifiers (left) and nine unsupervised classifiers (right).

CF(7)	1	2	3	4	5		CF(9)	1	2	3	4	5
**1**	84	82	80	32,554	76		**1**	84	82	76	80	32,554
**2**	0	6355	0	156	0		**2**	0	6355	0	0	156
**3**	0	0	7075	141	0		**3**	0	0	0	7075	141
**4**	7313	0	0	190	0		**4**	7313	0	0	0	190
**5**	0	0	0	135	6260		**5**	0	0	6260	0	135

**Table 3:** The results of the errors and number of found nodes using different values for ϵ and purity. The dimension of the blocks was 5.

Epsilon (ϵ)	Purity (p%)	Error	Number of Nodes
0.1	30	10.901%	2812
0.1	50	7.900%	6077
0.1	70	4.929%	17,642
0.1	90	3.685%	26,791
0.01	30	4.817%	18,933
0.01	50	3.407%	30,437
0.01	70	2.774%	37,938
0.01	90	2.732%	38,682
0.001	30	3.048%	35,332
0.001	50	2.798%	38,015
0.001	70	2.725%	38,787
0.001	90	2.725%	38,799
0.0001	30	2.786%	38,229
0.0001	50	2.747%	38,647
0.0001	70	2.725%	38,800
0.0001	90	2.725%	38,800
0.00001	30	2.759%	38,568
0.00001	50	2.743%	38,698
0.00001	70	2.725%	38,801
0.00001	90	2.725%	38,801

**Table 4:** Number of pixels (%) for each class for K-means, hierarchical and mean-shift clustering and silhouette score.

Data Set/Class	1	2	3	4	5	6	7	Silh. Score
Hierarchical (centroid)	11.11%	9.30%	12.88%	53.54%	13.16%			54.08%
Hierarchical (average)	11.11%	9.30%	12.88%	53.54%	13.16%			54.08%
Hierarchical (complete)	10.23%	34.01%	5.52%	16.88%	33.36%			65.16%
Hierarchical (ward)	14.68%	28.70%	15.41%	17.00%	24.21%			67.98%
K-means (K-means++)	18.73%	12.03%	25.86%	14.54%	28.84%			70.28%
K-means (random)	14.71%	12.21%	24.78%	19.31%	28.99%			70.16%
Mean-shift	30.91%	34.24%	10.32%	15.32%	9.21%			71.26%
K-means (random)	13.12%	12.57%	9.91%	16.78%	25.02%	22.61%		70.33%
K-means (random)	12.06%	11.08%	20.88%	18.48%	13.55%	9.45%	14.50%	72.62%

**Table 5:** The number of all pixels founded in each class.

Class	Water	Vegetation 1	Sand/Ground	Clouds	Vegetation 2
**Number**	9422 (15.57%)	24,754 (40.91%)	3655 (6.04%)	14,052 (23.22%)	8618 (14.24%)
